# Analyzing Unstructured Communication in a Computer-Mediated Environment for Adults With Type 2 Diabetes: A Research Protocol

**DOI:** 10.2196/resprot.7442

**Published:** 2017-04-24

**Authors:** Allison A Lewinski, Ruth A Anderson, Allison A Vorderstrasse, Edwin B Fisher, Wei Pan, Constance M Johnson

**Affiliations:** ^1^ Duke University School of Nursing Durham, NC United States; ^2^ School of Nursing University of North Carolina at Chapel Hill Chapel Hill, NC United States; ^3^ Gillings School of Global Public Health University of North Carolina at Chapel Hill Chapel Hill, NC United States; ^4^ Peers for Progress Gillings School of Global Public Health University of North Carolina at Chapel Hill Chapel Hill, NC United States; ^5^ School of Nursing The University of Texas Health Science Center at Houston Houston, TX United States

**Keywords:** diabetes type 2, social support, adults, Internet, peer support, self-management, mixed methods, social interaction, secondary analysis

## Abstract

**Background:**

Individuals with type 2 diabetes have an increased risk for comorbidities such as heart disease, lower limb amputations, stroke, and renal failure. Multiple factors influence development of complications in a person living with type 2 diabetes; however, an individual’s self-management behaviors may delay the onset of, or lessen the severity of, these complications. Social support provides personal, informal advice and knowledge that helps individuals initiate and sustain self-management and adherence.

**Objective:**

Our aim was to gain an understanding of type 2 diabetes social interaction in a virtual environment, one type of computer-mediated environment (CME), and the social support characteristics that increase and sustain self-management in adults living with chronic illness.

**Methods:**

This study is a secondary analysis of longitudinal data collected in a CME study, Second Life Impacts Diabetes Education & Self-Management (1R21-LM010727-01). This virtual environment replicated a real-life community where 6 months of naturalistic synchronous voice conversations, emails, and text chats were recorded among participants and providers. This analysis uses a mixed-methods approach to explore and compare qualitative and quantitative findings. This analysis is guided by two theories: Strong/Weak Ties Theory and Social Penetration Theory. Qualitative data will be analyzed using content analysis, and we will complete descriptive statistics on the quantified variables (eg, average number of ties). Institutional review board approval was obtained in June 2016.

**Results:**

This study is in progress.

**Conclusions:**

Interventions provided through virtual environments are a promising solution to increasing self-management practices. However, little is known of the depth, breadth, and quality of social support that is exchanged and how interaction supports self-management and relates to health outcomes. This study will provide knowledge that will help guide clinical practice and policy to enhance social support for chronic illness via the Internet.

## Introduction

Type 2 diabetes (T2D) affects 9.3% of the adult population in the United States and is the 7^th^ leading cause of death [1]. The Centers for Disease Control and Prevention estimates an additional 8.1 million US adults are living with T2D, yet remain undiagnosed [1]. Complications of T2D include renal failure, lower limb amputations, and heart disease [1]; such complications are associated with an individual’s self-management behaviors [2-4]. Regular preventative care is also essential to preventing comorbidities and maintaining a baseline level of health [5]. However, no greater than 75% of adults report receiving standard, recommended T2D preventive care, such as vaccinations, annual eye exams, at least an annual glycosylated hemoglobin (HbA1c) test, and regular foot examinations [1]. Due to the increasing incidence and prevalence of T2D, health care providers and researchers need to explore innovative, accessible, and lower cost ways to enhance the self-management skills of those living with T2D [6,7].

Self-management of T2D is person-specific, ever-present, and dynamic [2], as individuals with T2D provide 99% of their own self-care [8]. Thus, daily disease management of T2D depends on an individual’s self-management behaviors and knowledge [2]. In addition to T2D specific skills and knowledge, research indicates that psychosocial support is important in maintaining self-management behaviors [9,10]. As such, interventions that provide additional support to facilitate self-management are essential, as individuals living with T2D report not receiving desired psychosocial support from close family and peers [11].

### Background

Individuals living with T2D can become knowledgeable about self-management behaviors and living with a chronic illness through social interactions with peers and providers [12]. Studies indicate that sustained support from peers and providers for T2D self-management is effective in lowering HbA1c levels because it reinforces critical self-management skills [7,10,13]. Table 1 provides definitions for terms utilized in this study [14-24].

**Table 1:** Terms used in this study.

Term	Definition for this study
Self-management behaviors	Daily activities completed by the individual living with T2D, which may include [14-16]: monitoring dietary intake, checking blood glucose values, medication adherence, physical activity, foot care
Social interaction	A bidirectional, verbal, or written exchange between two or more individuals on a mutually shared, central topic [17-19]
Social support	Personal, informal advice and knowledge that help individuals initiate and sustain T2D self-management behaviors, thus increasing adherence [20-22]
Computer-mediated environment	A computer medium that mediates communication among individuals [23,24]: email, discussion forums, text messaging, virtual environments

Self-management of T2D improves with increased frequency of social interaction [25-27]. Research indicates that high frequency interaction, over an extended period of time, with peers or health care professionals can impact and change self-management strategies and blood glucose levels in T2D interventions [6]. Social interaction is important in self-management as individuals provide real-world assistance to those with T2D. Key to this relationship is the mutual understanding of the shared experience of living with T2D [22,28-30]. Individuals may exchange support as well as obtain information from others during these synchronous and asynchronous interactions [20,31,32]. Social interactions enable individuals to verbalize this acquired knowledge [33,34].

Of note, an individual’s behavior can be influenced due to a social interaction [31,35]. Verbalizing a personal narrative influences self-management skills, emotional expression, health outcomes, and social support; the language used denotes an individual’s perspective and meaning of these situations [32,36-40]. However, frequent levels of interaction and support are not always feasible in traditional health care settings due to the temporal and financial constraints on both the individuals and provider [6,41,42]. Thus, using the Internet for social interaction is a potential solution and can include interaction among peers as well as between the individuals and provider.

Internet interventions are more widely accessible than other forms of health care [43]. Individuals access information and interact with an online community of support [44,45] and gain quality information to aid in the self-management of T2D [44-46]. Therefore, interventions provided via computer-mediated environments (CMEs) are a promising solution to increase self-management practices [47,48]. Current CME interventions to improve T2D self-management include mHealth [45,49], programs via the Internet [45,49], and telemonitors [50]. In CMEs, individuals gain information and benefit from being present with others [51]; their participation is both active (present, talking) and passive (present, not talking). Information gleaned through these online interactions supplements and enhances real-world knowledge, processes, and experiences [52-54].

Despite obtaining useful self-management skills and knowledge, attrition in T2D self-management programs remains a concern. Reasons for attrition include barriers that may be temporal (eg, working full- or part-time, scheduling conflicts), geographical (eg, distance to program), emotional (eg, apathy, priority of self-management), or technological (eg, engagement, computer problems) [25,55,56]. Internet interventions can address many of these barriers to attendance, thus potentially decreasing attrition in self-management interventions. Unfortunately, the rates of T2D self-management remain suboptimal, and Internet intervention studies to improve self-management report inconsistent findings in both short- and long-term effectiveness and sustainability [3,26,49,57,58]. Mixed results may stem from not having synchronous conversations in social interaction, which would provide sufficient depth and breadth in social support or not having the ability to obtain this type of support at convenient times [16].

A virtual environment focusing on T2D-specific self-management skills may influence the real-time social interactions among individuals and the support exchanged [16]. Virtual environments mimic real-world environments. The virtual environment is exploratory, interactive, extensive, and users can ultimately determine their own personal involvement and investment [59]. This computer-generated environment provides an illusion of the real world through a multisensory, interactive encounter, in which users feel *presence* and *co-presence* [60,61]. Presence, the feeling of being “there” in the environment, makes it feel as if the actions in the CME were occurring in the real world, and the user is completely engaged in the CME [60-62]. Co-presence is the feeling that others are present in the virtual environment and that one is in an interactive environment where interpersonal relationships can be initiated, formed, and maintained [60,61]. While virtual environments can have many different types of representations (eg, small towns, space crafts) and facilitate a variety of interactions, the proposed study describes a virtual environment as one that recreates a small town using three-dimensional graphics [61]. The replication of real-life environments can foster skills that promote real-world application of essential self-management behaviors [16].

Individuals self-represent as avatars within these environments, a type of CME, to receive both informal and formal learning opportunities, thus reinforcing positive T2D self-management techniques [46,61,63]. Avatars, when high in *agency* (eg, accurate representation of a person in real life) and *behavioral realism* (eg, degree to which objects in the virtual environment act like they do in the real world), increase the involvement and engagement of individuals in the virtual environment [61]. This real-time interaction and support may positively influence self-management skills and behaviors. However, a gap in knowledge exists regarding the characteristics of social interaction and social support exchanged among adults living with diabetes that contribute to sustained behavior change and self-management [64,65].

Thus, this current study will provide insight into the depth, breadth, and quality of the social interaction and social support exchanged in a virtual environment through the study of conversations among participants in combination with survey responses, health outcomes (HbA1c, body mass index), and activity data. The parent study, Second Life Impacts Diabetes Education and Self-Management (SLIDES; 1R21-LM010727-01), provided self-management support and education in a virtual environment, where all voice, email, and text-chat conversations were recorded in real-time over a 6-month period [16]. The knowledge generated from this study will help determine what features are important to include in future T2D self-management interventions and how to best facilitate high-quality, effective support. Here we describe the theoretical and analytic approaches to this study exploring the characteristics of social interaction and social support exchanged among adults living with T2D who interacted in a virtual environment.

### Theoretical Framework

**Figure 1:**
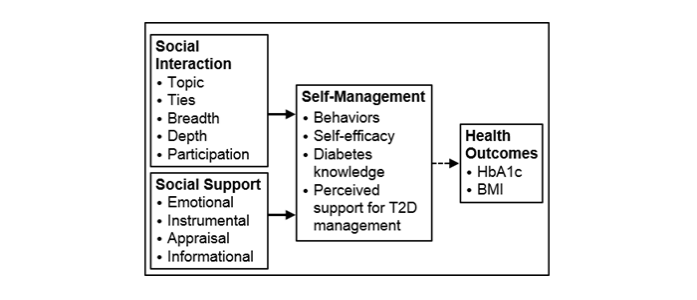
Guiding framework for this secondary analysis of qualitative and quantitative data from adults living with type 2 diabetes who interacted in a virtual environment. The bolded lines indicate the focus of this secondary analysis.

This study uses Social Penetration Theory [66] and Strong/Weak Ties Theory [67,68] to guide this secondary analysis in order to gain an understanding of T2D-specific social interaction among providers and peers within a CME. These theories will help us examine the differences in interaction among both active and passive participants, amount and type of interaction, and exchange of social support, specifically centering on self-management of chronic illness. Figure 1 depicts the guiding framework for this study.

#### Social Interaction

Social Penetration Theory suggests that the perceived value of a relationship influences the perceived rewards of initiating and maintaining participation, which then influences the *breadth* (eg, number of topics discussed) and *depth* (eg, degree of intimacy and personalization of discussed topics). Thus, increased breadth and depth will occur in a high reward (eg, family member, valued and trusted friend) relationship; low breadth and depth will occur in relationships that are considered low reward (eg, casual acquaintance) [66,69]. The determination of the value in a relationship influences the tie strength between two individuals.

In Granovetter’s [67] Strong/Weak Ties Theory, tie strength is time in which the relationship can develop and occur, intensity of emotions within that relationship, breadth and depth of intimacy, and whether the relationship is reciprocal and mutual. Strong and weak ties each serve a purpose. Strong ties provide support and intimacy, and weak ties provide linkages to information and resources outside of an individual’s circle of intimate relationships [67-69]. The number and type of topics discussed within these ties, such as social support, have the potential to influence tie strength. Strong ties are defined by closeness of contact, duration of contact, frequency of contact, and a direct link between two individuals. In sum, strong ties are densely connected [67-69] and the amount of social support is high. For instance, strong ties are an individual’s close family and friends with whom they frequently interact, and the ties are familiar with each other [68]. Weak ties are not densely connected, the relationships do not require large amounts of time or investment and can be formed more rapidly [67-69], and the amount and type of social support is lower than with strong ties. Weak ties are essential to a group; new information is diffused and shared between individuals [67-69]. An individual has weak ties with other people with whom they are not in frequent contact, and there is little familiarity among the individual’s other weak ties [68]. The characterization of the ties between individuals is important, as well as the identification of when ties are integral and influential in self-management and the exchange of support.

#### Social Support

We conceptualize social support as emotional, instrumental, informational, and appraisal support [70-74]. Emotional support is the provision of empathy shared among peers and providers when discussing T2D self-management. Instrumental support is the provision of assistance or goods that assist in the self-management of T2D. Informational support is the sharing of knowledge that assists the individual in T2D self-management. Appraisal support includes affirmational comments among individuals regarding self-management actions taken [75]. Peer support (eg, support from peers living with T2D) consists of assistance in daily management, linkage to clinical care, and the ongoing availability of support [29].

#### Self-Management and Health Outcomes

In this study, we utilized the outcome measures from the parent study to conceptualize self-management and health outcomes [16,48]. Data collected included valid and reliable measurement of (1) self-management behaviors via the Summary of Diabetes Self-Care Activities [76], (2) diabetes knowledge via true/false items designed to assess diabetes knowledge [77], (3) perceived support for T2D management via the Diabetes Support Scale [78], (4) self-efficacy via the Diabetes Empowerment Scale-Short Form [79], (5) outcome data (HbA1c and body mass index) via medical chart reviews by the study coordinator, (6) demographics collected by the study coordinator, and (7) activity data (number of logins, time spent online) via participant activity in the SLIDES site [16,48]. These data were collected at baseline, 3 months, and 6 months and are described fully elsewhere [16,48].

### Study Protocol

The overall goal of this study is to gain an understanding of T2D social interaction in a virtual environment, a type of CME, and the social support characteristics that increase and sustain self-management in adults living with this chronic illness. The specific aims for this study are:

- To describe the characteristics of social interaction using the following six *a priori* categories: (1) topics discussed, (2) strong/weak ties, (3) depth, (4) breadth, (5) participation, and (6) general engagement in the CME; and emergent codes that arise in a CME about self-management.
- To describe the characteristics of social support using the following four *a priori* categories: (1) emotional, (2) instrumental, (3) informational, and (4) appraisal; and emergent codes as they arise in a CME about self-management.
- To describe the trends of social interaction and social support over time, and the longitudinal relationship between social support and social interaction with SLIDES outcome data including self-management behaviors, self-efficacy, diabetes knowledge, perceived support for T2D management, health outcomes (HbA1c, body mass index), and participation (number of logins, time spent online).

### Design

A mixed-methods secondary analysis of naturalistic, conversational, qualitative data (voice and text conversations) will be used to describe the characteristics of social interaction in a CME about self-management and support [80-82]. This secondary analysis was approved by the University Institutional Review Board (Pro00022132).

### Parent Study

The SLIDES study looked at a virtual three-dimensional diabetes community that promoted knowledge application of self-management behaviors among adults with T2D [16,48]. The SLIDES sample included individuals living with T2D who self-represented as avatars (eg, representations of themselves) and interacted with peers while learning and practicing self-management skills. Individuals interacted in real-time self-management education and support classes focused on American Association of Diabetes Educators curriculum for self-management education and salient T2D self-management topics.

### Participants

All participants (N=20) and providers (diabetes educators and study investigators) (N=4) of the SLIDES study, and all conversations among participants, will be used. No further recruitment of participants will occur, and no additional inclusion or exclusion criteria will be applied. The demographics and primary outcomes of this study have been previously reported elsewhere [16,48]. As we want to analyze the various ways that individuals participated, we will include all 20 participants knowing that some participated more actively than others. Passive and active participation are described in the operationalization of social interaction. This allows us to understand what is happening to those individuals who are more/less active and more/less passive. The qualitative conversation data have not been analyzed in the parent study.

### Data Preparation

An Institutional Review Board‒approved transcriptionist and the first author transcribed the synchronous voice conversations. The first author verified concordance with the MP3 voice conversation files to ensure the accuracy of spoken words and the communication style of each participant. Each spoken word is linked to a SLIDES participant (de-identified), location of conversation in the SLIDES CME, and calendar date of conversation and is organized into a Microsoft Word file. Since the participants were provided a study-created screen name, their personal names were de-identified. These data were then organized by study week and uploaded into Atlas.ti for analysis.

### Measures

#### Qualitative Data

These data include all synchronous voice conversations and asynchronous conversations (eg, text chat, discussion forums, and emails) over 6 months (1535 pages of transcribed text), thus providing secondary data for evaluating social interaction [16,48]. Table 2 provides the operationalization of social interaction [66-69,83] and social support [70-74] for this study. Conversations occurred in various contexts: participant to participant, participant to educator, discussion forums, and within group education and support sessions [16,48].

#### Quantitative Data

We will be using data that were previously collected and analyzed in the parent study [16,48]. Demographic data were collected on entry into the SLIDES study, and activity data were collected continuously when the participants entered the SLIDES site.

### Data Analysis Plan

#### Overview

Table 3 provides a description of the analysis plan for the qualitative aims. The qualitative aims, characterizing social interaction and characterizing social support, will be first analyzed with content analysis [84] using Atlas.ti to manage and support the coding process.

**Table 2:** Operationalization of social interaction, social support, and source of support for this secondary analysis.

Variable	Operationalization in this study
Social interaction	Topic [83]: the content of the discussion about self-management, T2D, or living with chronic illness
	Ties (strong/weak) [67,68]: amount and duration of contact, intensity of emotions, reciprocity of interaction
	Depth [66,69]: degree of intimacy and personalization of discussed topics
	Breadth [66,69]: number of topics discussed
	Participation (active/passive): present and talking; present, not talking
Social support (4 categories as noted in the literature [70-74])	Emotional: exchange of feelings of trust, caring, love, belongingness, and warmth when discussing T2D self-management or behaviors
	Instrumental: exchange of tangible goods or services related to T2D self-management
	Informational: exchange of T2D-specific information among individuals
	Appraisal: exchange of praise for a T2D self-management behavior or action
	Emergent codes: to capture instances in the conversations not covered by the 4 categories of social support
Source of support (providers of support and/or education within the CME)	Provider: nurse practitioners, certified diabetes educator, principal investigator of SLIDES
	Peer: another individual with T2D

**Table 3:** Data analysis plan for the qualitative aims.

Aim and steps	Plan	
Study Aim 1: Characterizing social interaction	To describe the characteristics of social interaction using six a priori categories: (1) topics discussed, (2) strong/weak ties, (3) depth, (4) breadth, (5) participation, (6) general engagement in the CME; and emergent codes that arise in a CME about self-management	
Study Aim 2: Characterizing social support	To describe the characteristics of social support using the four a priori categories of social support: (1) emotional, (2) instrumental, (3) informational, and (4) appraisal; and emergent codes as they arise in a CME about self-management	
Analysis Step 1: First level coding process (data-near coding process)	Determine appropriate coding unit for each a priori code	
	Demographic coding (eg, conversation type, participant ID, participant study time, location in virtual environment, class type, conversation type)	
	Code data:	
		Social interaction: Use a priori codes (Table 1)
		Social support: Use a priori codes (Table 1)
	Team process: Code independently, gather together and debate definitions and coding, re-code documents following the meeting	
Analysis Step 2: Second level coding process (increasing abstraction of codes)	When themes are created, create variables	
	Create higher level, more abstract codes based on the first level codes: Social interaction and Social support	
Team process: Same steps taken as in the first level coding process	

**Table 4:** Data analysis plan for the mixed-method aim.

Aim and steps	Plan
Study Aim 3: Mixed-methods aim	To describe the trends of social interaction and social support over time, and the longitudinal relationship between social support and social interaction with SLIDES outcome data including self-management behaviors, self-efficacy, diabetes knowledge, perceived support for T2D management, physiological data (HbA1c, body mass index), and activity data (number of logins, time spent online)
Analysis Step 3: mixing the data (identifying areas of convergence and divergence of these data)	Identify patterns that emerge that can be described with sample demographics (eg, race, duration of diabetes)

The codes and themes created using content analysis will be quantitized into numerical values in order to create code counts for use in displaying trajectory lines for the mixed-method aim [85,86]. Table 4 provides the analysis plan for the mixed-methods aim. Emergent codes will be identified in relation to observations of social support unique to participants living with T2D interacting in a CME. Codes will be analyzed consistent with the theoretical framework and in the context of social interaction, social support, and self-management of T2D. Thus, the theoretical framework provides the lens to examine the conversations with a more focused and guided analysis.

#### Validity and Rigor

We will use team coding procedures to ensure validity and reliability of findings and iteratively generate codes based on the theories [86,87]. We will ensure validity by providing rich descriptions of all codes with exemplar quotations, triangulating data from quantitative and qualitative sources, presenting any discrepant information identified during the coding process, and discussing all findings as a team [88]. Validity of findings is also strengthened due to the extensive time the first author (AAL) spent cleaning and organizing these data, the involvement of the last author (CMJ) who served as Principal Investigator of SLIDES, and the participation of the third author (AAV), a co-investigator in SLIDES who led the support sessions [88]. A codebook will be created that details creation of the codes and emerging themes and that contains an audit trail of actions throughout coding and analysis [89]. The coding team (AAL, RAA, CMJ) will meet regularly to ensure accuracy of coding, reliability, categorization, higher level code development, and emerging findings. The coding team will independently read and code 25% of the transcripts during the entire analysis to ensure reliability.

#### Qualitative Aims: First Level Coding

The coding team will initially independently code the transcripts using the a priori codes for 5% of the transcripts and then meet to discuss codes and coding units. The team will compare examples and findings and discuss results until agreement is reached on coding definitions and application of the definitions to these data. The coding team will look for coding agreement for the remaining 5% of cycles of coding. Due to the multidimensional structure that is anticipated to be present in the transcripts, no limit will be placed on the number of codes to which a coding unit can be assigned. The coding team will repeat this process until we have agreement on the first level coding. Following discussions, the first author will re-code transcripts using any new codes, and the coding team will review work on a bi-weekly basis.

#### Qualitative Aims: Second Level Coding

We will use data matrices to identify patterns in these data that emerge relating to social interaction and social support [90]. The coding team will meet to identify higher level and more refined codes, inclusive of the codes identified in first level coding. Second level coding will mirror the first level coding in that the coding team will independently code 5% of the transcripts and then meet to discuss emerging patterns in these data. No limit will be placed on the number of higher level codes that a coding unit can be assigned. These data, and the patterns identified, will be quantitized to variables related to social interaction and social support (eg, levels of depth, active listening) [85]. Descriptive statistics will be used to summarize differences, determine frequencies, and identify relationships.

#### Mixed-Methods Aim

**Figure 2:**
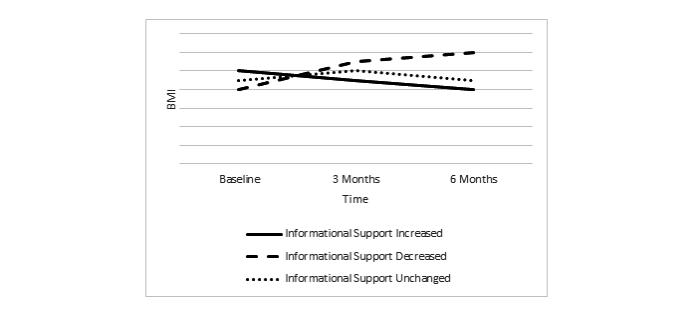
Sample of graphs for Aim 3, the mixed-methods aim for BMI and time. We first visually categorize the 20 trajectories of informational support into increased, unchanged, and decreased groups. Here shows a plot of the three subgroup’s BMI across the time points to see if the trajectories of BMI are correlated with the informational support: BMI decreased for the group of increased information support (filled line), BMI unchanged for the group of unchanged informational support (dotted line), and BMI increased for the group of decreased informational support (dashed line).

The SAS program version 9.4 will be used to address this aim. Due to the small sample size (N=20), we will compute descriptive statistics only to summarize the characteristics of social interaction and social support. These data will then be used to examine the longitudinal relationship between these variables with body mass index and HbA1c. The variables created during analysis of these qualitative data for the social interaction and social support aims will be used in the analysis of the mixed-methods aim. These data will be first plotted on trajectory lines for each person for the social interaction and social support variables (eg, ties, emotional support), and two or three subgroups will be identified by visually examining their trends over time (eg, informational support increased, unchanged, or decreased). Then we will summarize the average trend for each variable created from qualitative data (eg, ties, emotional support) and overlay the SLIDES variables (eg, diabetes knowledge, self-efficacy) for the subgroups to identify the trajectories at three time points (ie, baseline, 3 months, 6 months) and relate these to body mass index and HbA1c. Figure 2 provides a sample of such graphs to visualize these data.

The visual and descriptive trajectory lines will allow us to overlay the average trends from the time points to describe trends over time and how social support and social interaction evolve. We will determine if there are differences in the trajectory plots described above, based on demographic factors, duration of diabetes, and time spent online.

## Discussion

### Principal Considerations

Research indicates that sustained social support reinforces T2D self-management behaviors [7,29,91,92]. Current T2D social support research focuses on face-to-face interactions and CMEs with person-to-person interaction via the Internet [7,29,47-49,91,92]; yet, interventions for T2D over the past 15 years have not led to significant long-term improvements in self-management [49]. Therefore, a need exists for sustainable, innovative interventions that increase an individual’s self-management behaviors by providing long-term contact with providers and peers that provide T2D specific support [93-95].

Type 2 diabetes self-management is positively influenced by sustained, continuous support via face-to-face or Internet environments [26,49]. Research shows that relationships formed in CMEs augment face-to-face support; however, specifics of these relationships remain unknown [96,97]. Thus, we do not have sufficient evidence for how to encourage and support effective interactions in CMEs. Sufficient knowledge about interaction in CMEs is needed as approximately 81% of all US adults use the Internet and 72% look for health information online [98-100]. Thus, the analysis of verbatim, naturalistic conversations in a virtual environment, will characterize enacted, supportive interactions among individuals living with a chronic illness who are seeking information and support. Results from this study will provide a way to measure social support as it is provided in daily conversation and interaction to identify the real-time exchange of support among adults living with chronic illness.

The SLIDES platform allowed participants to have conversations online and exchange support in naturalistic conversations. Interactions in virtual environments are synchronous and include sight (eg, one can see graphics), sound (eg, one can hear other individuals talking and other ambient sounds in the environment), voice (eg, one can talk to others via a headset), text (eg, text-chatting with another person), and motion (eg, one can direct their avatar and navigate around the CME) [61,101]. This synchronous communication and the feelings of presence and co-presence mimic the real-time communication that occurs in relationships in the real world. Our belief is that conversational depth and breadth will occur in the virtual environment because individuals will feel like they are in the virtual environment (presence), with others (co-presence), and in a real-life conversation with another person and not an avatar (synchronous communication) [61]. Analysis of these naturalistic conversations will determine if the four categories of social support are reflected in social interactions in a CME. To our knowledge, this is the first study to analyze and characterize (1) social interaction among participants interacting in a T2D CME, in order to understand the nature of this kind of social interaction, and (2) social support in naturalistic conversations, in order to understand how to improve the exchange and delivery of social support in T2D specific interventions to populations with high rates of T2D. This data-rich sample allows for the identification of the nature of T2D-specific social support and social interaction in a CME using descriptive analysis and trajectory creation.

### Limitations

Limitations of this study include the small sample size (N=20), having only one male participant, and the inability to probe individuals to clarify statements. These factors limit the generalizability of the findings, and thus findings will be interpreted with caution. However, a sample of this size will enable us to analyze the conversations among individuals in depth, so that we can fully understand the phenomenon of interaction in a CME [102]. The value of analyzing naturalistic conversations will aid in understanding the nature of social support and social interaction in a CME and its benefits for self-management. With these data, we hope to see instances of social support that are rich in personal narratives, describe living with T2D, and contain emotional connections with others.

### Conclusions

The current study is unique because in the parent study, all interaction occurred within the virtual environment, which mimicked real life. The proposed study will determine the type of social support being exchanged in natural conversation through social interaction within a virtual environment by participants, and the subsequent changes in self-management. These results could be used to develop sustainable self-management interventions that promote high-frequency support. The proposed study will lead to further research to validate the findings in other populations. Subsequent research will aid in the development of effective and scalable self-management interventions that can reach large numbers of individuals including disadvantaged or diverse groups. The proposed study is a conceptual step in the development of self-management interventions aimed at improving population-level prevention and management of T2D, thus addressing the population burden and disparities seen with this chronic illness.

## Abbreviations

CME: computer-mediated environment
HbA1c: glycosylated hemoglobin
SLIDES: Second Life Impacts Diabetes Education and Self-Management (1R21-LM010727-01)
T2D: type 2 diabetes

## Appendix

Multimedia Appendix 1 Resume and summary of discussion from the NIH-NINR grant review process.
