# Supplementary material for: Analyzing Unstructured Communication in a Computer-Mediated Environment for Adults With Type 2 Diabetes: A Research Protocol
Source: JMIR Res Protoc. 2017 Apr 24;6(4):e65. doi: 10.2196/resprot.7442 (PMC5422658; doi:10.2196/resprot.7442)
Supplement: Multimedia Appendix 1 [file resprot_v6i4e65_app1.pdf]

1F31NR016622-01 Lewinski, Allison

RESUME AND SUMMARY OF DISCUSSION: This report summarizes the initial scientific merit review of a new application for a Ruth L. Kirschstein National Research Service Award (NRSA) for Individual Predoctoral Fellows in Nursing Research submitted by Ms. Allison Lewinski, who requests 18 months of support for her Ph.D. study. Using the mixed method design with secondary data collected for sponsor's prior R21 study, the applicant plans to describe the social interaction in computer mediated environments (CMEs), and the social/peer support characteristics that increase and sustain self-management behaviors for participants having type-2 diabetes (T2D). This application comes from a highly qualified candidate as evidenced with strong clinical and research experience, several publications and presentations, good graduate grades, several small funding, and excellent letters of recommendation. The sponsoring team including a sponsor, a cosponsor, and several collaborators are outstanding in terms of the match of scientific and methodological contents, current funding, and mentoring experience. Studying the potential importance of CME providing social and peer support to increase T2D patient self-management is highly needed and proposed mixed method study is appropriate. Utilization of the existing dataset from the parent R21 is both innovative and largely feasible. Research and training environment is excellent. Several minor weaknesses are mainly related to the research and training plan. The proposed training activities are not well linked with the training goals. The included theories have some mix-up for several potential mediators; it is unclear how to integrate the qualitative and quantitative date; and the included sample has its limitation in generalization of the finding. Overall, the strengths in the outstanding candidate, fully capable sponsoring team, potentially significant study, and superb environment far outweigh those minor weaknesses. This application has been ranked at the outstanding level for its scientific and technical merit.

DESCRIPTION (provided by applicant): Significance: Type-2 diabetes (T2D) is the 7th leading cause of death in the U.S., as patients with T2D are at increased risk for comorbidities such as heart disease, lower limb amputations, stroke, and renal failure. Type- 2 diabetes and its complications depend largely on a patient's own self-management behaviors, as regular preventative care is essential to preventing comorbidities and maintaining a baseline level of health. Improving self-management of T2D is imperative with 9.3% of U.S. adults diagnosed and an estimated 8.1 million people who remain undiagnosed. As the incidence and prevalence of T2D increase each year, healthcare providers examine how to increase a patient's self-management skills. Social and peer support provides personal, informal advice and knowledge that helps individuals initiate and sustain T2D self-management behaviors, thus increasing adherence to these behaviors. Specifically, current T2D social/peer support research focuses on face-to-face interactions, computer mediated environments (CMEs) with person-to-person interaction via the Internet, mobile health, and the telephone. In CMEs, social interaction is a verbal or written exchange between two or more individuals on a mutually shared, central topic. Interventions provided through CMEs are a promising solution to increase self-management practices' however, little is known about the social interactions among individuals in CMEs, specifically the frequency and content of the social/peer support that is exchanged and how social interaction supports self-management. Purpose: The overall purpose of this study is to gain a comprehensive understanding of social interaction in CMEs, and the social/peer support characteristics that increase and sustain self-management behaviors. Methods: This secondary analysis, using a mixed methods approach, will use data previously collected in a CME study, Second Life Impacts Diabetes Education & Self-Management (SLIDES, 1R21-LM010727-01, PI: Johnson). A convergent parallel mixed method design will be used to explore and compare the qualitative and quantitative findings. The SLIDES site was a virtual replication of a real life community in which 6-months of naturalistic synchronous voice conversations were recorded among participants and providers (500 pages of transcribed text). Summary: This study will increase understanding of social interaction in CMEs, specifically how social/peer support is spontaneously exchanged among individuals living with T2D. Knowledge gained from this proposal will help to guide clinical and public health practice and
